# Clinical and Proteomic Insights into a Cytokine Release Syndrome Triggered by Tebentafusp in a Metastatic Uveal Melanoma Patient: Case Report

**DOI:** 10.3390/jcm14041333

**Published:** 2025-02-17

**Authors:** Antonio David Lazaro Sanchez, Javier David Benitez Fuentes, Ginés Luengo Gil, María Teresa García García, Eduardo Feliciangeli Moreno, Pablo Conesa Zamora, José Balsalobre Yago, Kauzar Mohamed Mohamed, Ana Belén Arroyo Rodríguez

**Affiliations:** 1 Department of Medical Oncology, Morales Meseguer General University Hospital, 30008 Murcia, Spain; 2 Laboratory Medicine and Pathology Department, Group of Molecular Pathology and Pharmacogenetics, Biomedical Research Institute from Murcia (IMIB), Santa Lucía General University Hospital, 30202 Cartagena, Spain; gluengo@ucam.edu (G.L.G.); pconesa@ucam.edu (P.C.Z.); anabelen.arroyo@um.es (A.B.A.R.); 3 Department of Medical Oncology, Elche General University Hospital, 03203 Alicante, Spain; javierdavidbenitezfuentes@gmail.com; 4 Health Sciences Faculty, Universidad Católica de Murcia (UCAM), 30107 Guadalupe, Spain; tggarc@gmail.com (M.T.G.G.); edufeliciangeli@gmail.com (E.F.M.); josebalsalobreyago@gmail.com (J.B.Y.); 5 Department of Medical Oncology, Santa Lucia General University Hospital, 30202 Cartagena, Spain; 6 Department of Immunology, IML and IdISSC, Hospital Clinico San Carlos, 28040 Madrid, Spain; kauki96@gmail.com

**Keywords:** cytokine release syndrome, uveal melanoma, immunotherapy, proteomic analysis

## Abstract

**Background:** Uveal melanoma is the most common primary intraocular cancer in adults; however, it remains rare. Despite its rarity, metastatic uveal melanoma poses significant treatment challenges. Tebentafusp, a T-cell receptor–bispecific molecule targeting glycoprotein 100 and CD3, has shown substantial survival benefits for HLA-A*02:01 positive patients. A notable complication associated with tebentafusp and similar immunotherapies is cytokine release syndrome (CRS), occurring in nearly 90% of tebentafusp-treated patients. Although typically mild, severe CRS (grade 3) affects around 1% of patients. The unpredictable nature of CRS complicates patient management during treatment. **Methods:** Monitoring cytokine levels, as key indicators of inflammation, may therefore be crucial for understanding and managing CRS. Advanced proteomic technologies enable the simultaneous measurement of multiple cytokines, providing a comprehensive view of inflammatory responses. **Results:** In this case, a patient with metastatic uveal melanoma developed CRS after tebentafusp treatment. A proteomic analysis tracked the cytokine changes from baseline to post-treatment, revealing significant elevations in inflammatory markers. **Conclusions:** These findings suggest potential strategies for more personalized CRS management in similar therapies.

## 1. Background

Uveal melanoma is the most common primary intraocular cancer in adults; however, it remains rare, with an incidence of 5.1 new cases per million people in the USA [1]. This malignancy primarily affects the choroid, ciliary body, and iris [2]. The management of uveal melanoma primarily involves local control through therapies such as plaque brachytherapy, proton beam therapy, or enucleation, depending on the tumor size and location. However, metastatic uveal melanoma, particularly to the liver, remains challenging due to its poor prognosis and limited treatment options. Current systemic therapies include immune checkpoint inhibitors and liver-directed treatments, which have shown modest efficacy [2]. Tebentafusp, an intravenously administered drug targeting glycoprotein 100 and CD3, has emerged as a promising option, showing substantial survival benefits for HLA-A*02:01 positive patients, with about 27% surviving at three years [3]. It functions as a T-cell receptor–bispecific molecule, exploiting the presence of this HLA subtype to engage T cells against uveal melanoma cells.

**Figure 1:**
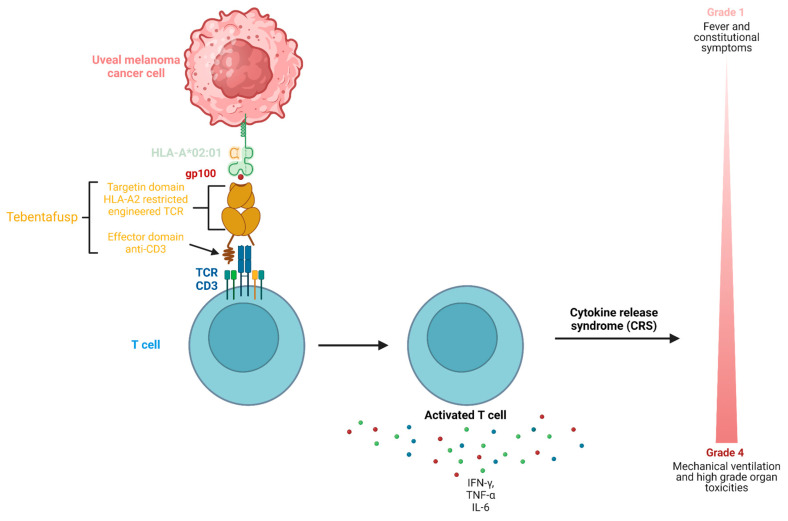
Illustration of the mechanism of action of tebentafusp.

A notable complication associated with tebentafusp and similar immunotherapies is cytokine release syndrome (CRS), occurring in nearly 90% of tebentafusp-treated patients (Figure 1). Although typically mild, severe CRS (grade 3) affects around 1% of patients [3]. Characterized by excessive cytokine release, CRS can lead to high fever and hypotension, complicating patient management. The unpredictable nature of CRS complicates patient management during treatment [4]. Monitoring cytokine levels, as key indicators of inflammation, may therefore be crucial for understanding and managing CRS [5,6].

Advanced proteomic technologies enable the simultaneous measurement of multiple cytokines, providing a comprehensive view of inflammatory responses. In this case, a patient with metastatic uveal melanoma developed CRS after tebentafusp treatment. A proteomic analysis tracked the cytokine changes from baseline to post-treatment, revealing significant elevations in inflammatory markers, suggesting potential strategies for more personalized CRS management in similar therapies.

## 2. Case Presentation

A 68-year-old Caucasian white woman with a history of hypertension, hyperuricemia, and hypothyroidism, which were all under control with medication, presented, in June 2021, with a diagnosis of choroidal melanoma in the anterior and superior temporal regions of the right eye’s posterior chamber. This diagnosis was confirmed by orbital magnetic resonance imaging (MRI), which also revealed choroidal detachment. No evidence of distant metastasis was found at that time. One month later, she underwent enucleation of the right eye with prosthetic reconstruction. Since the procedure, regular follow-ups with PET-CT scans have been conducted by the oncology and ophthalmology departments according to local follow-up guidelines.

**Figure 2:**
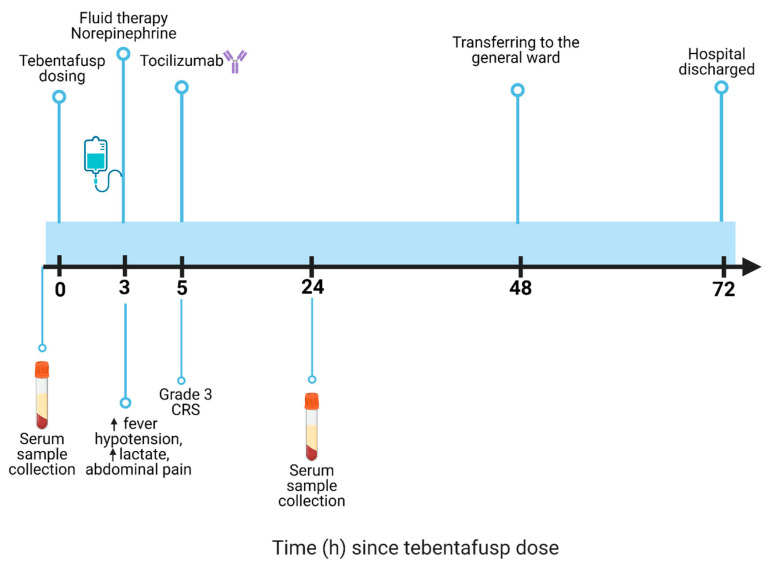
Timeline of events.

No recurrence was detected until three years later, when a follow-up PET-CT scan identified multiple metastases in the lungs, pleura, liver, and lymph nodes (internal mammary, bilateral hilar, and hepatic hilum). Genetic testing using PCR-based HLA typing confirmed the presence of HLA A02*01 positivity. Consequently, treatment with tebentafusp was proposed. In June 2024, the patient was admitted to the Intensive Care Unit for tebentafusp initiation as part of the local protocol. Figure 2 illustrates the timeline of events.

**Figure 3:**
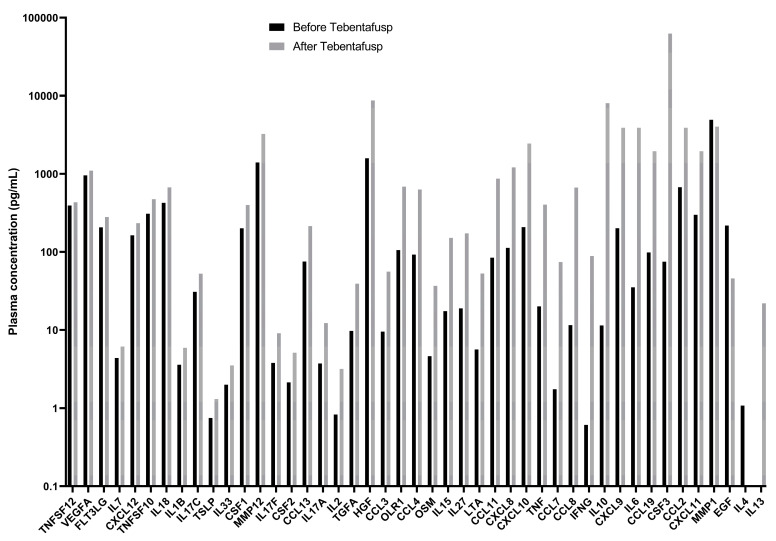
Graph showing the percentage change in cytokine levels before and after tebentafusp administration. C-C motif chemokine 3 (CCL3), C-C motif chemokine 4 (CCL4), C-C motif chemokine 19 (CCL19), C-X-C motif chemokine 9 (CXCL9), C-X-C motif chemokine 10 (CXCL10), C-X-C motif chemokine 11 (CXCL11), eotaxin (CCL11), FMS-related tyrosine kinase 3 ligand (FLT3LG), granulocyte colony-stimulating factor (CSF3), granulocyte–macrophage colony-stimulating factor (CSF2), hepatocyte growth factor (HGF), interferon gamma (IFNG), interleukin-1 beta (IL1B), interleukin-2 (IL2), interleukin-4 (IL4), interleukin-6 (IL6), interleukin-7 (IL7), interleukin-8 (CXCL8), interleukin-10 (IL10), interleukin-13 (IL13), interleukin-15 (IL15), interleukin-17A (IL17A), interleukin-17C (IL17C), interleukin-17F (IL17F), interleukin-18 (IL18), interleukin-27 (IL27), interleukin-33 (IL33), oxidized low-density lipoprotein receptor 1 (OLR1), macrophage colony-stimulating factor 1 (CSF1), macrophage metalloelastase (MMP12), interstitial collagenase (MMP1), C-C motif chemokine 2 (CCL2), C-C motif chemokine 7 (CCL7), C-C motif chemokine 8 (CCL8), C-C motif chemokine 13 (CCL13), oncostatin-M (OSM), pro-epidermal growth factor (EGF), stromal cell-derived factor 1 (CXCL12), thymic stromal lymphopoietin (TSLP), tumor necrosis factor ligand superfamily member 12 (TNFSF12), lymphotoxin-alpha (LTA), tumor necrosis factor ligand superfamily member 10 (TNFSF10), protransforming growth factor alpha (TGFA), tumor necrosis factor (TNF), and vascular endothelial growth factor A (VEGFA).

Plasma samples were collected prior to drug administration and 24 h later and were analyzed using a highly specific proteomic approach with the Olink^®^ Target 48 Cytokine reagent kit. This advanced kit employs a Proximity Extension Assay (PEA) technology to simultaneously measure 45 human protein biomarkers associated with inflammation, using only 1 µL of plasma. The PEA technology ensures exceptional specificity and sensitivity in detecting cytokines, allowing for accurate and reliable measurements even at low concentrations [7]. Notably, the Olink^®^ Target 48 Cytokine kit has been validated with healthy controls, and quantitative reference levels are well established, providing a robust benchmark for assessing cytokine levels. The detailed baseline values and 24 h post-tebentafus administration cytokine levels are provided in Figure 3.

Three hours post-administration, the patient developed a high fever (39.1 °C), hypotension (systolic BP of 60 mmHg), and elevated lactate levels (5.7 mmol/L). She experienced abdominal pain, which was managed with standard analgesia, and maintained an oxygen saturation of above 90% without respiratory distress. Despite the initial fluid therapy, her hypotension persisted, requiring IV norepinephrine starting at 0.02 μg/kg/min and increasing to 0.09 μg/kg/min. Due to grade 3 CRS, characterized by persistent hypotension needing vasopressors and elevated lactate, IV tocilizumab (8 mg/kg) was administered 19 h before the second plasma sample was collected, in accordance with clinical practice guidelines. The patient also showed signs of mild renal dysfunction with metabolic acidosis due to hypoperfusion and developed mild bilateral pleural effusion with passive atelectasis in the right lung base.

Following these interventions, her blood pressure stabilized, lactate levels normalized, diuresis was adequate, renal function improved, and capillary refill returned to normal. She remained conscious and alert throughout. On 21 June, once clinically stable and tolerating the cessation of vasopressors, she was transferred to the general ward for further monitoring.

The patient was discharged on 22 June 2024. Due to the toxicity experienced with tebentafusp, it was decided that she would proceed with second-line therapy consisting of ipilimumab (3 mg/kg) and nivolumab (1 mg/kg).

## 3. Discussion

This case highlights the challenges of treating metastatic uveal melanoma with tebentafusp in HLA-A*02:01-positive patients. While tebentafusp offers substantial survival benefits, it also carries significant risks, particularly CRS [8]. In our patient, CRS required prompt and effective management, underscoring the importance of timely intervention to prevent severe complications. This situation also demonstrates the potential utility of advanced cytokine profiling, as the detailed analysis provided insights into the inflammatory response, suggesting possible applications for predicting and managing CRS in similar treatments going forward.

A proteomic analysis of cytokine levels provided valuable insights into the inflammatory response. Elevated baseline cytokine levels, with 33 of the 45 cytokines raised, suggested a pre-existing pro-inflammatory state that may have contributed to the severity of CRS following tebentafusp administration. Among the elevated cytokines were VEGFA, FLT3LG, IL7, IL18, IL1B, IL17C, TSLP, IL33, CSF1, MMP12, IL17F, CSF2, IL17A, IL2, TGFA, HGF, CCL3, OLR1, IL15, IL27, CXCL8, TNF, CCL7, CCL8, IFNG, IL10, CXCL9, IL6, CCL2, CXCL11, MMP1, EGF, and IL4. Elevated baseline levels of cytokines in melanoma patients have been mainly studied for IL-6 and IL-10, with elevated initial levels of IL-10 being associated with increased mortality, while a progressive increase in IL-6 predicts a worse prognosis in melanoma. IL-10 acts as an immunosuppressant, inhibiting T-cell and macrophage activity, which facilitates immune evasion by tumor cells and promotes tumor progression. On the other hand, IL-6 plays a crucial role in chronic inflammation, favoring a pro-inflammatory tumor microenvironment that contributes to cell proliferation and survival, as well as metastasis. However, for other cytokines, such as those mentioned in our study, no evidence documenting elevated baseline levels in patients before specific cancer treatment has been found [9]. Plasma samples collected before and after tebentafusp administration revealed marked elevations in several cytokines, including CCL11, CXCL8, CXCL10, TNF, CCL7, CCL8, IFNG, and IL10. Notably, CXCL9, IL6, CCL19, CSF3, CCL2, and CXCL11, which were elevated to such extreme levels that they exceeded the assay’s quantification capacity, resulting in overflow readings and preventing the precise measurement of these heightened concentrations. The pronounced elevation of CXCL9 and CCL2, chemokines that recruit T cells and monocytes to sites of inflammation, might have amplified the immune response and contributed to the severity of the CRS [5,10,11,12,13]. IL-6, a central cytokine in CRS pathophysiology, promotes acute phase responses, and its high levels are associated with CRS severity, making it a primary target for the therapy used in this case, tocilizumab [5,10,11,13]. CCL19 is involved in the migration of dendritic cells and T cells to lymphoid tissues, and its elevation may indicate the enhanced mobilization of immune cells, contributing to the CRS [13]. CSF3 stimulates the production of white blood cells, with increased levels suggesting the increase in leukocyte production, potentially exacerbating CRS symptoms [12]. CXCL11 attracts activated T cells and is involved in skin immune responses, with its significant increase indicating a chemotactic response for T cells, further amplifying the immune response [5,10,14]. Some of these cytokines may not only indicate the severity of CRS but also serve as potential therapeutic targets, similar to how IL-6 is currently targeted in CRS treatment. Notably, two other markers, MMP1 and EGF, decreased from elevated baseline values to normal levels in the second test. MMP1 is a matrix metalloproteinase involved in degrading extracellular matrix components and regulating vascular integrity. Elevated MMP1 levels are often upregulated in metastatic cancers, including uveal melanoma, where they are associated with an aggressive tumor phenotype and poor prognosis. In addition to their role in tumor progression, elevated MMP1 levels can disrupt endothelial barriers, contributing to inflammatory responses and tissue damage, as observed in conditions like sepsis and potentially in CRS [14,15,16,17]. EGF, an important growth factor, plays a role in cell growth, proliferation, and differentiation. The overexpression of EGF or dysregulation of its receptor (EGFR) pathway has been implicated in the progression of many cancers, including uveal melanoma [18,19]. The normalization of MMP1 and EGF levels post-treatment may reflect a rebalancing of inflammatory signals in response to the therapy with tebentafusp.

In a study that enrolled 84 patients with metastatic melanoma treated with tebentafusp, with 19 being metastatic uveal melanoma, Middleton et al. tested cytokine levels in 13 of the metastatic uveal melanoma patients [15]. They reported similar findings of elevated cytokines post-treatment and highlighted the importance of cytokine monitoring in managing CRS [15]. The study emphasized that the inflammatory response involves multiple cytokines, including CXCL10, CXCL9, and IFNγ, which were also elevated in our study [15].

Cytokine testing has been explored as a method to predict CRS in other settings. Diorio et al. performed comprehensive serum proteome profiling in patients with B-cell acute lymphoblastic leukemia (B-ALL) receiving CART19 therapy and identified significant elevations in cytokines such as IFNγ, which plays a fundamental role in CRS [6]. They identified MILR1 and FLT3 as pre-infusion biomarkers predictive of severe CRS and noted that CRS may be an IFNγ-driven process with a protein signature overlapping with hemophagocytic lymphohistiocytosis [6]. While our study highlighted elevations in CCL11, CXCL8, and IL6, Diorio et al. focused on IFNγ and its related proteins [6]. In a cohort of patients with acute lymphoblastic leukemia treated with CAR-T, Teachey et al. revealed that a signature composed of IFNG, sgp130, and sIL1RA could predict severe CRS [18]. As previously mentioned, we, similarly, found that IFNG was elevated in our case [18]. Wei et al. also explored biomarkers for predicting severe CRS in B-ALL patients treated with CAR-T therapy [17]. They identified peak levels of cytokines, including IFNγ, IL6, and IL10, as being highly associated with severe CRS, which is consistent with our findings [19]. However, specific research on the prediction of CRS in T diseases, such as T-lymphoblastic leukemia, is limited. Therefore, although research in this area is ongoing, there is still a lack of sufficient evidence to support the use of cytokines as predictive biomarkers of CRS in patients with T diseases.

In several types of cancers, genetic polymorphisms have also been studied as potential biomarkers to predict CRS in patients treated with immune therapies. In lung cancer, genetic variants in cytokine genes, such as IL6, IFNG, and TGFβ1, have been found to be associated with an increased severity of CRS after treatment with PD-1 inhibitors, suggesting that these biomarkers may help to predict the exacerbated inflammatory response. Similarly, in leukemias such as B-ALL, polymorphisms in genes such as CD28 have also been linked to the intensity of CRS in patients receiving CAR-T cell therapy, an immunotherapeutic modality. In addition, variants in the TNFA gene (-308 G/A) and IL6 have been identified in various cancers, such as melanoma and other solid tumors, that increase the production of pro-inflammatory cytokines, which may predict the increased severity of CRS [20,21,22,23,24,25]. However, despite these findings in different cancer types, in the case of metastatic uveal melanoma, the evidence on specific genetic biomarkers for predicting CRS remains limited.

In the context of metastatic uveal melanoma, tebentafusp has established itself as the most effective first-line option for HLA-A*02:01-positive patients, given its significant impact on improving survival. Although the risk of CRS associated with its use is a clinical challenge, there is no therapeutic alternative that offers comparable results in this population. While second-line therapies, such as immune checkpoint inhibitors, targeted therapies, and combination strategies, have been explored, none have been shown to surpass the benefits of tebentafusp in terms of efficacy and survival, reaffirming its position in the standard management of these patients [26,27].

Identifying strong links between cytokine levels and CRS severity remains challenging. However, the early detection of CRS risk is crucial for improving patient safety and treatment outcomes. Our findings suggest that cytokine profiling could be a promising tool for assessing CRS risk in uveal melanoma patients treated with tebentafusp. Although preliminary, this approach could potentially reveal additional treatment targets beyond tocilizumab and, furthermore, may contribute to a better understanding of the pathophysiology of CRS, which is not yet fully understood.

## Data Availability

The original contributions presented in this study are included in the article. Further inquiries can be directed to the corresponding author.

## References

[1] Singh A.D., Bergman L., Seregard S. (2005). Uveal melanoma: Epidemiologic aspects. Ophthalmol. Clin. N. Am..

[2] Jager M.J., Shields C.L., Cebulla C.M., Abdel-Rahman M.H., Grossniklaus H.E., Stern M.H., Carvajal R.D., Belfort R.N., Jia R., Shields J.A. (2020). Uveal melanoma. Nat. Rev. Dis. Primers.

[3] Hassel J.C., Piperno-Neumann S., Rutkowski P., Baurain J.-F., Schlaak M., Butler M.O., Sullivan R.J., Dummer R., Kirkwood J.M., Orloff M. (2023). Three-Year Overall Survival with Tebentafusp in Metastatic Uveal Melanoma. N. Engl. J. Med..

[4] Acharya U.H., Dhawale T., Yun S., Jacobson C.A., Chavez J.C., Ramos J.D., Appelbaum J., Maloney D.G. (2019). Management of cytokine release syndrome and neurotoxicity in chimeric antigen receptor (CAR) T cell therapy. Expert Rev Hematol..

[5] Morris E.C., Neelapu S.S., Giavridis T., Sadelain M. (2022). Cytokine release syndrome and associated neurotoxicity in cancer immunotherapy. Nat. Rev. Immunol..

[6] Diorio C., Shraim R., Myers R., Behrens E.M., Canna S., Bassiri H., Aplenc R., Burudpakdee C., Chen F., DiNofia A.M. (2022). Comprehensive Serum Proteome Profiling of Cytokine Release Syndrome and Immune Effector Cell-Associated Neurotoxicity Syndrome Patients with B-Cell ALL Receiving CAR T19. Clin. Cancer Res..

[7] Lundberg M., Eriksson A., Tran B., Assarsson E., Fredriksson S. (2011). Homogeneous antibody-based proximity extension assays provide sensitive and specific detection of low-abundant proteins in human blood. Nucleic Acids Res..

[8] Shah D., Soper B., Shopland L. (2023). Cytokine release syndrome and cancer immunotherapies—Historical challenges and promising futures. Front. Immunol..

[9] Martínez-Escribano J.A., Campillo J.A., Piñero A., Frías J.F., Sánchez-Pedreño P., Corbalán R., Minguela A., Rocío Alvarez M. (2005). Prospective study of the levels of serum cytokines in patients with melanoma: Prognostic value. Actas Dermosifiliogr..

[10] Leclercq-Cohen G., Steinhoff N., Albertí Servera L., Nassiri S., Danilin S., Piccione E., Yángüez E., Hüsser T., Herter S., Schmeing S. (2023). Dissecting the Mechanisms Underlying the Cytokine Release Syndrome (CRS) Mediated by T-Cell Bispecific Antibodies. Clin. Cancer Res..

[11] Tvedt T.H.A., Vo A.K., Bruserud Ø., Reikvam H. (2021). Cytokine Release Syndrome in the Immunotherapy of Hematological Malignancies: The Biology behind and Possible Clinical Consequences. J. Clin. Med..

[12] Tsioumpekou M., Krijgsman D., Leusen J.H.W., Olofsen P.A. (2023). The Role of Cytokines in Neutrophil Development, Tissue Homing, Function and Plasticity in Health and Disease. Cells.

[13] Lei W., Zhao A., Liu H., Yang C., Wei C., Guo S., Chen Z., Guo Q., Li L., Zhao M. (2024). Safety and feasibility of anti-CD19 CAR T cells expressing inducible IL-7 and CCL19 in patients with relapsed or refractory large B-cell lymphoma. Cell Discov..

[14] Jiang Y., Tsoi L.C., Billi A.C., Ward N.L., Harms P.W., Zeng C., Maverakis E., Kahlenberg J.M., Gudjonsson J.E. (2020). Cytokinocytes: The diverse contribution of keratinocytes to immune responses in skin. JCI Insight.

[15] Middleton M.R., McAlpine C., Woodcock V.K., Corrie P., Infante J.R., Steven N.M., Evans T.R.J., Anthoney A., Shoushtari A.N., Hamid O. (2020). Tebentafusp, A TCR/Anti-CD3 Bispecific Fusion Protein Targeting gp100, Potently Activated Antitumor Immune Responses in Patients with Metastatic Melanoma. Clin. Cancer Res..

[16] Tressel S.L., Kaneider N.C., Kasuda S., Foley C., Koukos G., Austin K., Agarwal A., Covic L., Opal S.M., Kuliopulos A. (2011). A matrix metalloprotease-PAR1 system regulates vascular integrity, systemic inflammation and death in sepsis. EMBO Mol. Med..

[17] Wang T., Zhang Y., Bai J., Xue Y., Peng Q. (2021). MMP1 and MMP9 are potential prognostic biomarkers and targets for uveal melanoma. BMC Cancer.

[18] Teachey D.T., Lacey S.F., Shaw P.A., Melenhorst J.J., Maude S.L., Frey N., Pequignot E., Gonzalez V.E., Chen F., Finklestein J. (2016). Identification of Predictive Biomarkers for Cytokine Release Syndrome after Chimeric Antigen Receptor T-cell Therapy for Acute Lymphoblastic Leukemia. Cancer Discov..

[19] Wei Z., Xu J., Zhao C., Zhang M., Xu N., Kang L., Lou X., Yu L., Feng W. (2023). Prediction of severe CRS and determination of biomarkers in B cell-acute lymphoblastic leukemia treated with CAR-T cells. Front. Immunol..

[20] Sade-Feldman M., Jiao Y.J., Chen J.H., Rooney M.S., Barzily-Rokni M., Eliane J.-P., Bjorgaard S.L., Hammond M.R., Vitzthum H., Blackmon S.M. (2017). Resistance to checkpoint blockade therapy through inactivation of antigen presentation. Nat. Commun..

[21] Fitzgerald J.C., Weiss S.L., Maude S.L., Barrett D.M., Lacey S.F., Melenhorst J.J., Shaw P., Berg R.A., June C.H., Porter D.L. (2017). Cytokine Release Syndrome After Chimeric Antigen Receptor T Cell Therapy for Acute Lymphoblastic Leukemia. Crit. Care Med..

[22] Ceschi A., Noseda R., Palin K., Verhamme K. (2020). Immune Checkpoint Inhibitor-Related Cytokine Release Syndrome: Analysis of WHO Global Pharmacovigilance Database. Front. Pharmacol..

[23] Sepesi B., Ye Y., Mitchell K.G., Zhang L., Gu J., Ji L., Antonoff M.B., Hofstetter W.L., Rice D.C., Mehran R.J. (2018). Genetic variants in cytokine signaling pathways and clinical outcomes in early-stage lung cancer patients. J. Thorac. Cardiovasc. Surg..

[24] Hirano T. (2021). IL-6 in inflammation, autoimmunity and cancer. Int. Immunol..

[25] Michot J.M., Bigenwald C., Champiat S., Collins M., Carbonnel F., Postel-Vinay S., Berdelou A., Varga A., Bahleda R., Hollebecque A. (2016). Immune-related adverse events with immune checkpoint blockade: A comprehensive review. Eur. J. Cancer.

[26] Algazi A.P., Tsai K.K., Shoushtari A.N., Munhoz R.R., Eroglu Z., Piulats J.M., Ott P.A., Johnson D.B., Hwang J., Daud A.I. (2016). Clinical outcomes in metastatic uveal melanoma treated with PD-1 and PD-L1 antibodies. Cancer.

[27] Carvajal R.D., Schwartz G.K., Tezel T., Marr B., Francis J.H., Nathan P.D. (2017). Metastatic disease from uveal melanoma: Treatment options and future prospects. Br. J. Ophthalmol..

